# Anticancer, Antitumor, and Antioxidant Microbial Lipids: Mechanism of Action, Production, and Clinical Application

**DOI:** 10.1155/omcl/5525367

**Published:** 2026-06-11

**Authors:** Morteza Nadeb, Seyed Soheil Aghaei, Tahereh Komeili-Movahed, Hamed Afkhami

**Affiliations:** ^1^ Department of Microbiology, Qo.C., Islamic Azad University, Qom, Iran, qom-iau.ac.ir; ^2^ Applied Physiology Research Center, Qom Medical Sciences, Islamic Azad University, Qom, Iran, qom-iau.ac.ir; ^3^ Cellular and Molecular Research Center, Qom University of Medical Sciences, Qom, Iran, muq.ac.ir

**Keywords:** anticancer, antioxidant, antitumor, clinical applications, microbial lipids, polyunsaturated fatty acids

## Abstract

The present document highlights the significance of microbial lipids as new generation anticancer, antitumor, and antioxidant compounds. It focuses on the diverse nature of the action of these lipids in cancer treatment, especially polyunsaturated fatty acids (PUFAs). These mechanisms include membrane disruption, apoptosis/necrosis, DNA damage modifying histone and gene expression, angiogenesis inhibition, cell proliferation/cycle regulation, migration, invasion, metastasis, differentiation, reversal of drug resistance, and immune system modulation. The text also discusses the economical biosynthesis of these lipids via microbial fermentation, chemosynthesis, and other synthesis techniques. The versatility of PUFAs is described in detail in the following areas of study including signal transduction and inflammation. While microbial lipids offer potential cost‐effectiveness due to scalable fermentation processes, it is important to note that specific clinical dosage regimens and comprehensive pharmacoeconomic comparisons with standard chemotherapeutic agents are still under investigation. Current estimates suggest lower long‐term costs if optimized dosing is achieved, but further clinical trials are required to validate these economic benefits.

## 1. Introduction

Cancer ranks as one of the top killers which caused nearly 10 million deaths in 2020, and 6 out of 100 deaths globally. Cancer remains a global problem, the prevalence of which increases constantly and creates considerable burdens for healthcare systems and societies [1]. Standard treatments for this ailment have included chemotherapy and radiation therapy has also played a crucial role in cancer treatment. However, these therapies bear with them severe side effects [2]. For example, fatigue affects 87% of chemotherapy patients, followed by weight loss at 71.4% and diarrhea at 49.4% [3]. They may also experience other early side effects of radiotherapy: skin changes and tiredness, which are quite temporary but can affect a person’s day‐to‐day life greatly [4].

For this reason, there is need and an increasing demand to find other forms of treatment that could be effective and have lesser side effects. Among them the most prospective is the use of polyunsaturated fatty acids (PUFAs), omega‐3 and omega‐6 PUFAs in particular as they have some evidence of anticancer activity [5]. Research paper indicate that PUFAs has the ability to cause cancer cells to die, inhibit the growth of tumor cells and disrupt their functioning. Remarkably, they can modify the membrane fluidity and porosity, thereby affecting signal transduction that is critical for the cancer cells [6].

Moreover, on account of their ability to decrease the levels of free radicals and reduce oxidative DNA damage, lipid, and protein damage, PUFAs are implicated in cancer protection [7]. Studies have also shown interaction of PUFAs with other apoptotic markers in pancreatic cancer cells, and the possibility of utilizing ferroptosis in other resistant types of cancers the current scenario and future prospects of cancer therapy presents a wide area of possible therapeutic targets for PUFA mediated therapies for cancer.

**Figure 1:**
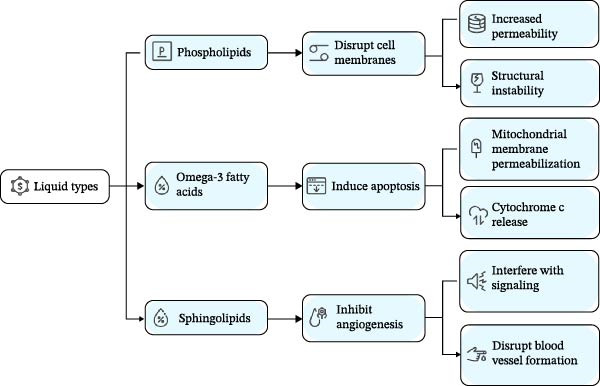
Mechanisms of microbial lipids in cancer therapy: structural insights and cellular interactions. This schematic illustrates the multifaceted anticancer mechanisms of microbial lipids, particularly PUFAs. The figure depicts how these bioactive lipids integrate into cancer cell membranes, altering membrane fluidity and permeability, which subsequently disrupts critical signaling pathways. Key mechanisms shown include (1): membrane disruption leading to calcium homeostasis imbalance, (2) induction of apoptosis through mitochondrial pathways and caspase activation, (3) generation of reactive oxygen species (ROS) causing oxidative stress, (4) DNA damage and histone modifications affecting gene expression, (5) inhibition of angiogenesis via VEGF downregulation, and (6) modulation of immune responses. The diagram emphasizes the selective toxicity of microbial lipids toward cancer cells while sparing normal cells, highlighting their therapeutic potential as multi‐target anticancer agents with lower toxicity profiles compared to conventional chemotherapy.

Microbial lipids particularly PUFAs are emerging as a new generation of anticancer agents. These are as follows: ability to induce apoptosis, suppression of cell division, and interference with cancer cell metabolism. For example, some of the PUFAs interfere with cell membrane fluidity and permeability, which interferes with signaling that is important for cancer cells growth [8]. Also, the antioxidant effect of PUFAs plays a role in cancer prevention through elimination of free radicals and inhibition of oxidative DNA, lipid, and protein damage [9, 10] (Figure 1).

This is particularly important because oxidative stress is central to the development of cancer.

Global cancer rates are increasing and so is cancer death, therefore there is a need to develop new and effective interventions [11]. Though conventional anticancer agents such as chemotherapy and radiation therapy have proved useful, they have a number of drawbacks including severe toxicity, drug resistance, and high cost [12]. These challenges are even more acute in the areas with scarce healthcare facilities and economic problems and in this connection, it is necessary to find other, less toxic, and cheaper therapeutic assets [13].

This review is based on a narrative synthesis of current literature retrieved from major scientific databases including PubMed, Scopus, and Web of Science, focusing on studies investigating microbial lipid‐mediated anticancer mechanisms and production strategies.

The article is aimed at providing comprehensive information on anticancer, antitumor, and antioxidant microbial lipids on a particular emphasis on PUFAs as an important group of these lipids. It includes their mechanisms of action, production and clinical applications (Table 1). The article particularly discusses the possibility of PUFAs as cheap and low toxicity therapeutic options for cancer, especially in areas with low income.

**Table 1:** Comparison of microbial lipids vs. traditional cancer treatments.

Feature	Microbial lipids	Traditional cancer treatments	References
Mechanism of action	Potentially strike target specific pathways with a great deal of precision resulting in less damage to healthy cells	It is often nonspecific and affects both healthy and cancerous cells	[14, 15]
Side effects	Potentially less and less severe side effects	Side effects such as hair loss, nausea, etc	[16]
Cost‐effectiveness	They could be more cost effective in the long run if lower doses, and less frequent dosing are used	Complex manufacturing processes and administration needs, typically high cost	[17, 18]
Resistance development	Unique mechanisms of action that lower likelihood of resistance development	Resistance development high potential, necessitating change in treatment regimens	[19]
Application in therapy	They can be engineered for targeted delivery: increasing efficacy and decreasing systemic exposure	This has broad application, but rarely has the ability to target tumor cells alone	[20, 21]
Environmental impact	Often sustainable production methods are possible, with reduced environmental impact	The production and disposal processes can be highly environmentally demanding	[22]

Anticancer and antitumor actions of microbial lipids, including PUFAs, are also associated with the alteration of the sphingolipids that control death and survival signaling [23]. Therefore, due to the microbial fermentation’s ability to be scaled up and the low costs involved microbial lipid production including PUFAs can be made to occur in various places even the less developed countries [24, 25]. These developments allow for methods to enhance the productivity of lipid product in microbial cultures and functionality [24]. In this case, microbial lipids can be produced in areas that are not so economically developed hence minimizing the reliance on imported drugs [25].

The final forms of rigorous testing and validation that are required to take the advantages of microbial lipids from the laboratory to the clinic are ongoing. The effectiveness and toxicity of these compounds in human populations have not been evaluated, thus, calling for clinical trials to define their true value [26]. Furthermore, the delivery and therapeutic action of microbial lipids must be incorporated into suitable pharmaceutical forms such as capsules, injections or topical solutions [27].

Last but not least, microbial lipids, particularly PUFAs, represent an emerging and biologically versatile class of bioactive molecules with potential relevance in oncology. Their diverse mechanisms of action and feasibility of scalable production make them attractive candidates for further investigation, particularly in settings where cost‐effective therapeutic strategies are needed. However, substantial clinical validation is required before definitive therapeutic conclusions can be established [28]. Substances produced by these microorganisms are still considered as potential precursors for novel and more effective and at the same time more available antineoplastic agents [29]; however, much more research is required to make the best of these substances.

## 2. Mechanism of Action of Bioactive Microbial Lipids

### 2.1. Anticancer Effects of Microbial Lipids

Microbial lipids, particularly PUFAs such as phospholipids, sphingolipids, and fatty acids, exhibit significant anticancer properties through various mechanisms [30]. These bioactive lipids incorporate into cellular membranes, altering their structural and functional dynamics, which in turn modulate key signaling pathways essential for cancer cell proliferation and survival [31]. Additionally, microbial lipids promote apoptosis and induce cell cycle arrest, thereby inhibiting tumor growth. They also interfere with tumor metabolic processes and influence the immune response against cancer cells [32]. Furthermore, their antioxidant activity helps mitigate oxidative stress, a major contributor to cancer progression [33]. It is important to contextualize the mechanisms described in this section. While specific pathways such as ferroptosis induction or selective apoptosis are highlighted, these findings are predominantly supported by preclinical data. Methodological details, including specific cell lines, dosages, and exposure times, vary across the cited studies. Therefore, these mechanisms should be interpreted as promising preclinical evidence requiring further clinical validation. Through these diverse mechanisms, microbial lipids hold great potential for the development of novel anticancer therapies (Table 2).

**Table 2:** Anticancer activities of microbial lipids.

Microbial lipid	Microbial source	Targeted cancer type	Mechanism of action	References
Omega‐3 fatty acids	*Schizochytrium* sp., *Thraustochytrium* sp	Pancreatic cancer	Induces ferroptosis and inhibits proliferation via ROS generation	[34]
Polyunsaturated fatty acids (PUFAs)	*Crypthecodinium cohnii*, *Mortierella alpina*	Breast, colon cancer	Disrupts membrane fluidity, inhibits proliferation, and induces apoptosis	[35]
EPA (eicosapentaenoic acid)	Marine microorganisms (*Schizochytrium* sp.)	Prostate cancer	Modulates eicosanoid metabolism and reduces inflammatory signaling	[36]
DHA (docosahexaenoic acid)	Algal sources (*Thraustochytrium* sp.)	Breast cancer	Reduces angiogenesis and induces apoptosis via mitochondrial pathways	[37]
Monogalactosyldiacylglycerol	*Cyanobacteria*	Melanoma	Inhibits DNA polymerase activity and disrupts cancer cell replication	[38]
Beta‐Hydroxybutyrate	Produced during microbial fermentation	Colorectal cancer	Inhibits histone deacetylase, promoting apoptosis and reducing tumor growth	[39]
Sphingolipids	Yeasts like *Saccharomyces cerevisiae*	Lung cancer	Alters ceramide signaling pathways to induce cell death	[40]
Phosphatidylinositol	*Pseudomonas aeruginosa*	Ovarian cancer	Regulates signal transduction and inhibits cancer cell invasion	[41]
Alkylphospholipids	Synthetic derivatives	Liver cancer	Induces apoptosis and interferes with mitochondrial membrane integrity	[42]
Edelfosine	Synthetic analog of lysophosphatidylcholine	Leukemia, brain tumors	Induces apoptosis via Fas/CD95 pathway and inhibits MAPK/ERK signaling	[43]

*Note:* The anticancer activities and mechanisms listed are primarily derived from preclinical studies (in vitro cell lines or in vivo animal models). Specific experimental conditions, including cell line types, lipid concentrations, exposure durations, and assay methods, vary across the cited references and are not detailed in this summary table. Clinical evidence in human populations remains limited for most compounds, and further translational research is required to validate these mechanisms in clinical settings.

### 2.2. Antitumor Mechanisms of Microbial Lipids

Microbial lipids exhibit potent antitumor activity by targeting multiple hallmarks of cancer. They interfere with tumor cell signaling networks, disrupting communication essential for uncontrolled growth and survival [44]. By modulating lipid rafts in cellular membranes, they influence receptor‐mediated pathways that regulate tumor progression [45]. Additionally, microbial lipids alter the tumor microenvironment, reducing inflammation and inhibiting angiogenesis, which is crucial for tumor nourishment and expansion. Their role in metabolic reprogramming further weakens cancer cells by limiting essential nutrients and energy sources [46]. Readers should note that the antitumor activities summarized in Table 3 are largely based on experimental models. The specificity of these mechanisms, such as sparing normal cells while targeting tumors, has been observed primarily in controlled in vitro and in vivo settings. Variability in experimental design across studies necessitates cautious interpretation until robust clinical trials confirm these effects in human patients. These multifaceted actions position microbial lipids as promising agents in the fight against tumors (Table 3).

**Table 3:** Antitumor activities of microbial lipids.

Microbial lipid	Microbial source	Targeted tumor type	Mechanism of action	References
Sphingomyelin	*Saccharomyces cerevisiae*	Skin cancer	Regulates cell death via sphingolipid‐mediated signaling	[47]
Glycolipids	*Mycobacterium tuberculosis*	Lung cancer	Induces dendritic cell maturation, promoting antitumor immunity	[48]
Lipoteichoic acid	*Lactobacillus acidophilus*	Cervical tumors	Enhances immune responses and inhibits angiogenesis	[49]
Phosphatidylserine	*Bacillus subtilis*	Liver tumors	Modulates immune checkpoint activity and promotes apoptosis	[50]
Cardiolipin	*Escherichia coli*	Gastric tumors	Alters mitochondrial function to induce apoptosis	[51]
Trehalose dimycolate	*Mycobacterium* species	Lung tumors	Activates macrophages to kill tumor cells	[52]
Polyhydroxyalkanoates (PHA)	*Cupriavidus necator*	Colon cancer	Reduces tumor proliferation and modulates metabolic pathways	[53]
Lipopolysaccharides (LPS)	Gram‐negative bacteria	Skin tumors	Activates Toll‐like receptor signaling, enhancing immune‐mediated tumor regression	[54]
β‐Glucans	*Pleurotus ostreatus* (Oyster Mushroom)	Breast cancer (MCF‐7 cells)	Enhances immune responses and inhibits tumor cell proliferation	[55]
Alkyl‐Lysophospholipids (ALPs)	Synthetic analogs	Brain tumors	Selectively induces apoptosis in tumor cells while sparing normal cells	[56]

*Note:* The antitumor mechanisms summarized in this table are based on experimental data from preclinical models. Methodological details such as tumor models (xenograft, syngeneic, or spontaneous), dosing regimens, route of administration, and duration of treatment differ among the cited studies. Readers should interpret these findings as preliminary evidence supporting the therapeutic potential of microbial lipids, pending confirmation in well‐designed clinical trials.

### 2.3. Antioxidant Properties of Microbial Lipids

Microbial lipids play a crucial role in combating oxidative stress, a key contributor to cellular damage and disease progression [57]. They neutralize reactive oxygen species (ROS), preventing DNA damage, lipid peroxidation, and protein oxidation, which are linked to cancer and other chronic conditions. Additionally, these bioactive lipids enhance cellular antioxidant defense systems by upregulating protective enzymes and modulating redox‐sensitive signaling pathways [58]. Their ability to maintain cellular homeostasis and reduce oxidative stress makes them valuable candidates for therapeutic applications in disease prevention and treatment (Table 4).

**Table 4:** Antioxidant properties of microbial lipids.

Microbial lipid	Microbial source	Antioxidant properties	References
Polyunsaturated fatty acids (PUFAs)	*Mortierella alpina*, *Schizochytrium* sp	Eliminates free radicals, protects DNA from oxidative damage, and reduces lipid peroxidation	[59]
Tocopherols (Vitamin E‐like)	*Cyanobacteria*	Protects lipids and proteins from oxidative damage	[60]
Astaxanthin	*Haematococcus pluvialis*	Neutralizes ROS and protects against UV‐induced oxidative stress	[61]
Single cell oils (SCOs)	*Thraustochytrium* sp	Reduces oxidative stress in neuronal cells and inhibits lipid peroxidation	[62]
Phytosterols	*Rhizopus oryzae*	Inhibits free radical formation and enhances cellular antioxidant capacity	[63]
Docosahexaenoic acid (DHA)	*Schizochytrium* sp	Enhances antioxidant enzyme activity, reducing oxidative stress in cancer cells	[64]
Ergosterol	*Saccharomyces cerevisiae*	Scavenges ROS and protects mitochondrial integrity	[65]
Squalene	*Thraustochytrium* sp	Inhibits lipid peroxidation and enhances glutathione levels	[66]
Polysaccharide lipid complexes	*Aspergillus niger*	Protects cells from oxidative damage and reduces protein carbonylation	[67]
β‐Glucans polysaccharide	*Pleurotus ostreatus*	Scavenges ROS, reducing oxidative stress	[68]

### 2.4. Disruption of the Cell Membrane and Induction of Apoptosis/Necrosis

PUFAs, along with other microbial lipids such as the disruption of the cell membrane and the induction of apoptosis or necrosis of cancer cells via the action of microbial lipids, especially phospholipids and sphingolipids, represents a potent anticancer mechanism [69]. These lipids will integrate into the lipid bilayer of cancer cell membranes, and the physical and chemical properties of the membrane will be altered. This integration alters membrane fluidity and permeability, disrupting the ability of cancer cells to survive and proliferate and the signaling pathways that keep them that way [70].

An example of such a mechanism is the change of calcium homeostasis [71]. The concentration and distribution of calcium ions in cells (important for cellular function) can be inferred by microbial lipids. Causes of cell death in apoptosis or necrosis are due to an imbalance in calcium levels. Features of apoptosis, a programmed cell death, include chromatin condensation, membrane blebbing, and DNA fragmentation [72]. On the other hand, necrosis, which happens from acute cellular injury, leads to uncontrolled release of cellular contents with potential for inflammation [73] (Table 5).

**Table 5:** Factors of impaired cell membrane and induction of apoptosis/necrosis.

Cancer type	Factor	Mechanism	Example	Microbial source	References
Breast cancer	Lipopeptides	Disruption of cell membrane; induction of apoptosis; activation of signaling	Surfactin, Rakicidin	*Bacillus subtilis*, *Streptomyces* sp	[74]
	Polyunsaturated fatty acids	Increasing membrane fluidity; lipid peroxidation; inhibition of proliferation	Docosahexaenoic Acid (DHA), EPA	*Schizochytrium* sp., *Mortierella* sp	[75]
	Alpha‐galactosyl ceramides	Activation of Natural Killer T cells; apoptosis induction	KRN7000	*Sphingomonas* sp	[76]
Prostate cancer	Polyunsaturated fatty acids	Modulation of eicosanoid metabolism; reduced inflammatory signaling	Eicosapentaenoic Acid (EPA)	*Thraustochytrium* sp	[75]
	Fatty acid Potassium Salts	Induction of oxidative stress; disruption of mitochondrial function	FAPS from Nannochloropsis salina	*Nannochloropsis salina*	[77]
Skin cancer	Lipopeptides	Disruption of cell membrane; altering permeability; induction of apoptosis	Surfactin	*Bacillus subtilis*	[74]
	Alpha‐galactosyl ceramides	Activation of NKT cells; direct cancer cell attack	KRN7000	*Sphingomonas* sp	[76]
Colorectal cancer	Polyunsaturated fatty acids	Membrane fluidity increase; induction of apoptosis	Linoleic acid (LA), Gamma‐Linolenic Acid (GLA)	*Mortierella alpina*	[75]
	Fatty acid potassium salts	Disruption of mitochondrial function; oxidative stress induction	FAPS from *Thamnidium elegans*	*Thamnidium elegans*	[77]
Lung cancer	Lipopeptides	Disruption of cell membranes; apoptosis induction	Rakicidin	*Streptomyces* sp	[74]
	Polyunsaturated fatty acids	Lipid peroxidation; inhibition of cell proliferation	Arachidonic Acid (AA)	*Mortierella alpina*	[75]
Liver cancer	Polyunsaturated fatty acids	Induction of apoptosis; inhibition of cell growth	Docosahexaenoic Acid (DHA)	*Schizochytrium* sp	[75]
	Alpha‐galactosyl ceramides	Activation of NKT cells	KRN7000	*Sphingomonas* sp	[74]
Gastric cancer	Polyunsaturated fatty acids	Free radical production; inhibition of proliferation	Gamma‐Linolenic Acid (GLA)	*Mortierella alpina*	[75]
	Fatty acid potassium salts	Disruption of mitochondrial function; induction of oxidative stress	FAPS from Nannochloropsis salina	*Nannochloropsis salina*	[77]

*Note:* The factors and mechanisms of membrane disruption and apoptosis/necrosis induction presented here are compiled from various preclinical investigations. Experimental parameters, including lipid formulation, delivery method, cancer cell line characteristics, and assessment techniques (e.g., flow cytometry, Western blot, microscopy), are not uniformly reported across studies. These data highlight promising mechanistic insights but require standardization and clinical validation before therapeutic application.

**Figure 2:**
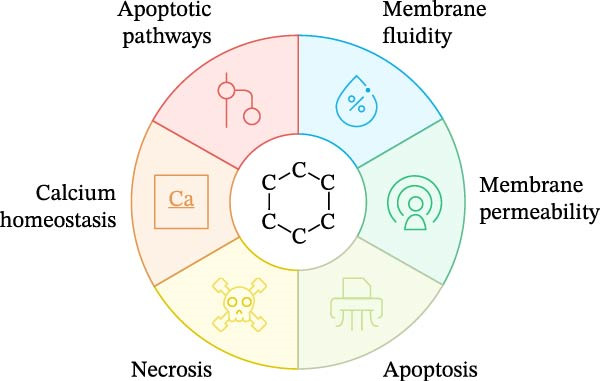
Microbial lipids and cell membrane dynamics: mechanisms of membrane disruption and cell death induction. This figure demonstrates the critical role of microbial lipids in disrupting cancer cell membrane integrity and inducing programmed cell death. The illustration shows how phospholipids, sphingolipids, and PUFAs incorporate into the lipid bilayer, causing: (A) increased membrane fluidity and permeability that compromises cellular integrity, (B) disruption of lipid raft domains affecting receptor‐mediated signaling, (C) alteration of calcium ion homeostasis triggering apoptotic cascades, and (D) activation of ceramide‐mediated death signaling pathways. The figure highlights the differential effects on cancer versus normal cells, showing how microbial lipids may preferentially affect malignant cells due to their altered membrane composition, as suggested by experimental studies. Additionally, the diagram illustrates the downstream consequences including chromatin condensation, membrane blebbing, DNA fragmentation (apoptosis), and uncontrolled cellular content release (necrosis), providing mechanistic insights into how membrane‐targeted therapy can effectively eliminate cancer cells.

In addition, it was found that sphingolipids, such as ceramides, also have a major role in mediating apoptotic pathways. Activation of protein kinases and phosphatases is induced by ceramides, which induce inhibition of survival pathway and activation of pro‐apoptotic signaling pathway [78]. These lipid molecules are involved in regulating key tumor cell signaling pathways, and their potential as targets for anticancer therapies that selectively cause tumor cell death is underscored [79]. Furthermore, the described mechanisms of membrane disruption and induction of cell death are supported by varying levels of experimental evidence. While some studies provide detailed methodological contexts regarding lipid concentrations and exposure durations, others offer broader mechanistic insights. Future research should aim to standardize these methodological parameters to better translate these findings into clinical applications. Consequently, the capacity of microbial lipids to disturb cell membrane integrity and regulate crucial apoptotic and necrotic pathways constitutes a promising strategy for anticancer treatments based on targeting the fundamental cellular processes required for tumor growth and survival [80] (Figure 2).

### 2.5. DNA‐Related Damages and Histone Modifications

Recently, microbial lipids, particularly PUFAs, have emerged as potent agents in oncology, as they are able to cause DNA related damage and induce histone modifications, which influence gene expression and cellular behavior in cancer cells [81]. We have shown that indirect oxidative DNA damage mediated through ROS generation and lipid peroxidation products, including sphingolipids and fatty acids, with the DNA can lead to oxidative damage, strand breakage, and impediment to replication and transcription processes necessary for cancer cell proliferation [82]. DNA damage is created by ROS or direct interaction to DNA structure resulting in adduct formation, cross‐linking, and base oxidation. These damages activate cellular DNA repair pathways, but when overwhelmed they result in cancer cell apoptosis or senescence [83].

In addition, microbial lipids regulate epigenetic regulation through modulation of histone modifications. Two HDACs, HDAC1 and HDAC3, and two HATs, p300 and CBP, can alter the activity of these enzymes [84]. For example, some microbial derived fatty acids have been shown to inhibit HDAC, increasing the level of histone acetylation. The hyperacetylation you get, these lead to this more relaxed chromatin state that allows for transcription of tumor suppressor genes, and blocks oncogene expression [85]. Induction of these epigenetic alterations by microbial lipids also increases the expression of genes that promote cell cycle arrest and apoptosis, as well as improves the cellular response to anticancer drugs [86]. As a result, microbial lipids possess a dual action on DNA integrity and histone modification, pointing to a major therapeutic avenue in cancer treatment, targeting key molecular pathways that are critical for tumor growth and survival [87] (Table 6).

**Table 6:** Microbial lipids in cancer therapy‐DNA damage and cellular effects.

Microbial lipid type	DNA damage induced	Resulting cellular effects	Microbial source	References
Sphingolipids	Induces DNA fragmentation	Apoptosis, cell cycle arrest	*Sphingomonas* sp., *Bacteroides* sp	[88]
Cardiolipins	Disruption of mitochondrial DNA	Apoptosis, mitochondrial dysfunction	*Rhodobacter sphaeroides*, *Bacillus* sp	[89]
Phosphatidylserine	Strand breaks, Forming adducts	Apoptosis, senescence	*Lactobacillus casei*, *Escherichia coli*	[90]
Phosphatidylethanolamine	Oxidative DNA damage	Necrosis, autophagy	*Escherichia coli*, *Pseudomonas* sp	[91]
Lipopeptides	Cross‐linking DNA	Apoptosis, immunogenic cell death	*Bacillus subtilis*, *Streptomyces* sp	[92]

### 2.6. Inhibition of Angiogenesis

Microbial lipids, particularly PUFAs such as omega‐3 fatty acids (e.g., EPA and DHA), play a key role in inhibiting angiogenesis, a fundamental process in tumor growth and metastasis [93]. Inhibiting angiogenesis, the fundamental process of tumor growth and metastasis in which new blood vessels form to supply nutrients to proliferating cancer cells, is a key role played by microbial lipids. Mechanisms through which this antiangiogenic action is mediated include alteration of signaling pathways and direct interaction with vascular endothelial cells [94]. In particular, microbial lipids including omega‐3 fatty acids have been found to reduce the expression of vascular endothelial growth factor (VEGF) and its receptors. VEGF is a major promoter of angiogenesis and its downregulation blocks a major part of the angiogenic cascade required for tumor vascularization [95]. As studies have shown, EPA and DHA, both omega‐3 fatty acids derived from microbial sources such as marine algae, reduce VEGF induced proliferation and migration of endothelial cells [96].

Moreover, microbial lipids may also modulate other angiogenic factors, including angiopoietins and matrix metalloproteinases (MMPs). They contribute to extracellular matrix structural remodeling, an important event for newly formed blood vessels. Microbial lipids inhibit MMP activity and thereby prevent basement membrane degradation and endothelial cell invasion and capillary formation [97].

Additionally, microbial lipids have anti‐inflammatory properties and those properties contribute to their antiangiogenic effects. Angiogenesis is closely associated with chronic inflammation and microbial lipids reduce cytokine production which in turn curtails an environment conducive to tumor angiogenesis [25, 98, 99].

Overall, microbial lipids interfere with angiogenesis by multiple means that act simultaneously to suppress key angiogenic mediators and receptors, compromise endothelial cell function, and reduce inflammation [100]. The comprehensive inhibition of angiogenesis by microbial lipids clearly indicates the potential of microbial lipids as therapeutic agents in cancer treatment, targeting both cancer cells directly and the supportive vascular networks that allow tumor growth and metastasis [101].

### 2.7. Interference With Cell Proliferation and Cell Cycle Regulation

Microbial lipids, including PUFAs and sphingolipids, are critical regulators of cell proliferation and cell cycle progression in cancer cells, offering potential therapeutic strategies against malignancies [102]. Critical regulators of cell proliferation and cell cycle progression in cancer cells have been identified as microbial lipids, which could provide potential therapeutic strategies against malignancies. Specific classes of these lipids (e.g., sphingolipids and fatty acids) function to modulate cell cycle checkpoints and signaling pathways important for controlling cellular growth and are responsible for these anticancer effects [103].

The regulation of the cyclin dependent kinase (CDK) inhibitors such as p21 and p27 is one primary mechanism by which microbial lipids control cell proliferation. Inhibition of CDKs by these inhibitors is essential to control cell cycle progression and to prevent transition from G1 to S phase of the cell cycle. For example, specific’ sphingolipids, such as ceramide, have been reported to upregulate p27^Kip1 expression, to inhibit cancer cell proliferation by promoting cell cycle arrest at the G1 phase [104].

More importantly, microbial lipids are able to activate different signaling pathways that induce apoptosis and restrict cell proliferation. For example, ceramide activates stress activated protein kinases (SAPKs), implicated in DNA damage and stress signal stimulatory pathways leading to programmed cell death [105] (Table 7).

**Table 7:** Microorganisms effective in interfering with cell proliferation and cell cycle regulation.

Microorganism	Type	Effective compound	Mechanism	References
*Bacillus subtilis*	Bacteria	Surfactin	‐ Induction of apoptosis via the ROS/JNK pathway and disruption of signaling in ERK1/2 and JNK pathways ‐ Induction of apoptosis by intracellular Ca2+ accumulation	[106]
*Micromonospora*	Bacteria	Rakicidin A and B	Induction of apoptosis in cancer cells	[107]
*Candida bombicola*	Yeast	Sophorolipids	Induction of apoptosis and cell cycle arrest in the G1 phase	[108]
*Nannochloropsis salina*	Microalgae	FAPS	Induction of apoptosis	[109]
*Thamnidium elegans*	Fungus	FAPS	Induction of apoptosis	[110]
Marine sponge	Porifera	Alpha‐galactosyl ceramides (KRN7000)	Activation of Natural Killer T (NKT) cells	[111]

In addition, the way in which microbial lipids interact with specific receptors and enzymes associated with cell growth and survival, such as the Akt/mTOR pathway, further identifies microbial lipids as proapoptotic agents to cancer cell proliferation. Inhibition of this pathway results in microbial lipids reducing protein synthesis and cell growth, thus improving anticancer efficacy [112].

**Figure 3:**
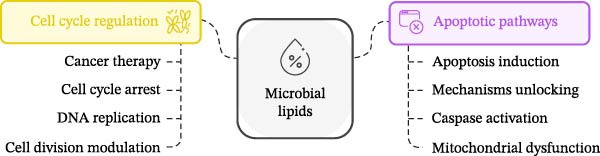
Impact of microbial lipids on cell cycle regulation and apoptotic pathways in cancer cells. This comprehensive diagram illustrates how microbial lipids interfere with cancer cell proliferation through dual mechanisms: cell cycle arrest and apoptosis induction. The figure depicts: (1) Upregulation of CDK inhibitors (p21, p27) by sphingolipids like ceramide, causing G1 phase arrest and preventing G1‐to‐S phase transition, (2) Activation of SAPKs leading to DNA damage responses, (3) Modulation of Akt/mTOR pathway inhibiting protein synthesis and cell growth, (4) Mitochondrial pathway activation with cytochrome c release and caspase cascade initiation, and (5) Regulation of Bcl‐2 family proteins shifting the balance toward pro‐apoptotic signaling. The illustration emphasizes the coordinated action of these pathways, showing how microbial lipids simultaneously halt cell division and trigger programmed cell death. This multi‐pronged approach demonstrates the therapeutic advantage of microbial lipids in overcoming cancer cell resistance mechanisms that often limit conventional single‐target therapies.

Overall, microbial lipids were found to inhibit cancer cell proliferation and control the cell cycle by modulating key CDK inhibitors, activating proapoptotic signaling pathway, and inhibiting major growth and survival pathways. The results of this study demonstrate that this multifaceted interference of microbial lipids with cell cycle and proliferation provides the basis for their use as effective agents in cancer therapy, targeting fundamental processes that are crucial for cancer cells to grow and survive [113] (Figure 3).

### 2.8. Inhibition of Migration, Invasion, and Metastasis

Microbial lipids, including PUFAs such as omega‐3 fatty acids and other lipids like sphingolipids, are potent inhibitors of cancer cell migration, invasion, and metastasis, critical stages in cancer progression and major sources of cancer morbidity and mortality [114]. Microbial lipids are critical stages in cancer progression and are major sources of cancer morbidity and mortality [115]. Other lipids, sphingolipids and specific fatty acids are antimetastatic not only by increasing cellular architecture but also by blocking key signal transduction pathways in the metastatic process [116]. Modifying the actin cytoskeleton is one of the major pathways by which microbial lipids exert their effects. The motility of cancer cells is critically dependent on changes in actin dynamics. For example, sphingolipids can alter from actin filaments organization, in order to prevent the formation of lamellipodia and filopodia, the critical morphologies for cell migration [117]. Microbial lipids also regulate the expression and function of MMPs. MMPs are enzymes which degrade components of extracellular matrix and contribute to the entry of cancer cells into the bloodstream via tissue barriers. By blocking the action of the MMPs, microbial lipids stop the degradation of extracellular matrix and confine the ability of cancer cells to invade [118].

Additionally, signal transduction pathways with relevance for cell adhesion and motility such as the focal adhesion kinase (FAK) and the PI3K/Akt pathways are under influence of microbial lipids. Cancer cells with inhibited pathways are less migratory and invasive [119].

In addition, some microbial lipids can adjust tumor microenvironment to be less permissive to metastasis. For instance, omega‐3 fatty acids have been demonstrated to reduce inflammation, an often linked phenomenon to increased tumor invasion and metastasis [120] (Table 8).

**Table 8:** Effect of lipids in inhibiting the migration, invasion, and metastasis of cancer cells.

Lipid type	Example	Mechanism	Microbial source	References
Lipopeptides	Surfactin	‐ Inhibition of MMP‐9, NF‐κB, and AP‐1 ‐ Inhibition of PI3K/Akt and MAPK signaling pathways ‐ Alterations in cell membrane fatty acid composition and induction of apoptosis	*Bacillus subtilis*	[121]
	Halobacillin	Moderate anti‐cancer activity	*Halobacillus* sp	[122]
	Mixirins A	Strong cytotoxic activity	*Streptomyces* sp	[123]
	Fengycin	Interaction with membrane lipids	*Bacillus subtilis*	[124]
	Pseudofactin II	Disruption of cell membrane integrity	*Pseudomonas fluorescens*	[125]
Fatty acids	PA	‐ Downregulation of STAT‐3 and Snail gene expression ‐ Downregulation of NF‐κB expression ‐ Increased expression of E, cadherin and decreased expression of vimentin	*Mortierella alpina*	[126]
	SM	‐ Downregulation of NF‐κB expression ‐Downregulation of Snail expression ‐ Inhibition of cancer cell proliferation and metastasis by reversing the polarization of M2 macrophages in tumors	*Sphingobacterium spiritivorum*	[127]
	DHA	‐ Downregulation of NF‐κB expression ‐ Downregulation of Snail and Stat3 expression ‐ Increased expression of E, cadherin and decreased expression of vimentin	*Schizochytrium* sp., *Thraustochytrium* sp	[128]
	Cer	‐ Downregulation of NF‐κB expression ‐ Downregulation of Snail and Stat3 expression	*Sphingomonas* sp	[129]
	Linoleic acid (LA)	Inhibition of intercellular communication via gap junctions (GJIC)	*Mortierella alpina*	[130]
	Gamma‐linolenic acid (GLA)	Reduced adhesion of breast and colon cancer cells to the endothelium by improving GJIC	*Mortierella* sp., *Schizochytrium* sp	[76]
Alpha‐galactosyl ceramides	KRN7000	Activation of Natural Killer T (NKT) cells that directly attack cancer cells and induce apoptosis	*Sphingomonas* sp	[111]
Fatty acid potassium salts (FAPS)	FAPS from *Nannochloropsis salina and Thamnidium elegans*	The exact mechanism is not clear, but it likely involves the induction of oxidative stress and disruption of mitochondrial function	*Nannochloropsis salina*, *Thamnidium elegans*	[131]

### 2.9. Induction of Differentiation

Microbial lipids, including PUFAs, sphingolipids (such as ceramide), and phospholipids (such as phosphatidylcholine and phosphatidylserine), show great potential for inducing differentiation in cancer cells, an important step for reprogramming malignant cells into less aggressive or more mature phenotypes and thereby decrease the cells proliferative capabilities [132]. In particular, these lipids, including sphingolipids and phospholipids, are involved in complex mechanisms to regulate cellular differentiation pathways, and thus represent potential therapeutic targets in oncology. Ceramide and other sphingolipids have been well studied for their ability to induce differentiation of different cancer cell types [133]. However, ceramide regulates the activity of key differentiation related signaling pathways, including the mitogen activated protein kinases (MAPKs) and protein kinase C (PKC), to promote differentiation. These pathways control the machinery used by the transcriptional machinery necessary for the induction and continuation of the differentiation process [134]. For example, the MAPK pathway frequently results in the activation of transcription factors essential to cell cycle arrest and differentiation.

In addition, other classes of microbial lipids, such as phosphatidylcholine and phosphatidylserine also play a role in cellular differentiation. The ability to alter the composition of cell membranes in a way that influences receptor function and downstream signaling cascades leading to differentiation, are these lipids. We have shown that phospholipids affect the integrity and localization of lipid rafts, cholesterol rich microdomains required for the clustering of signaling molecules and initiation of signaling events critical for differentiation [135].

Furthermore, microbial lipids can also modulate tumor microenvironment to support differentiation events in a secondary manner. These lipids modify inflammatory responses and interact with stromal cells to create conditions more favorable to differentiation than to proliferation [136].

In brief, microbial lipids induce differentiation of cancer cells by direct modulation of signaling pathways and transcription factors, changes in membrane dynamics, and influences on the tumor microenvironment [137]. The therapeutic potential of microbial lipids as agents that not only inhibit tumor growth but also reverse malignancy through differentiation is underscored by these mechanisms.

### 2.10. Reversal of Drug Resistance

Microbial lipids, including PUFAs, sphingolipids, and phospholipids, hold great promise for reversing drug resistance in cancer therapy, a major problem that often leads to treatment failure and disease progression. Multiple of these lipids, including sphingolipids and phospholipids, work through multiple mechanisms that back into the cellular processes and the pathways that contribute to drug resistance [138]. Modulation of drug efflux pumps, such as P‐glycoprotein (P‐gp), is one of the major mechanisms by which microbial lipids affect drug resistance. We have shown that some sphingolipids inhibit the activity of these pumps, raising the intracellular concentration of chemotherapeutic agents and improving their efficacy [139]. To take one example, cancer cells might be downregulated for expression of P‐gp, and drug efflux diminished, increasing cancer sensitivity to the drug [140].

Furthermore, microbial lipids may influence the lipid composition of cell membranes and thereby affect membrane fluidity and the function of membrane bound proteins such as drug transporters [141]. These lipids can disrupt membrane dynamics, preventing the localization and function of resistance related proteins, which can further sensitize cancer cells to chemotherapy [142]. Additionally, microbial lipids modulate apoptosis pathways that are often dysregulated in drug resistant cancer cells [143]. For example, ceramide has been shown to activate pro‐apoptotic signaling pathways including caspases and mitochondrial pathways to cause cell death that would otherwise be resistant to drug induced cell death.

**Figure 4:**
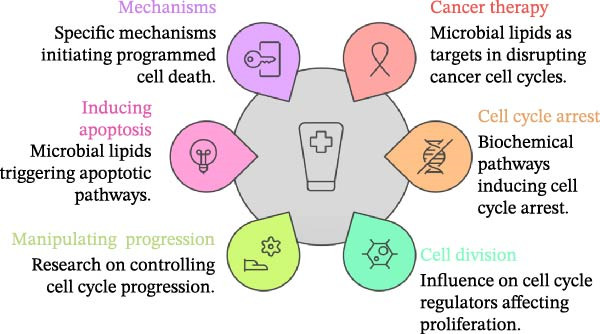
Comprehensive mechanisms of microbial lipids in cancer therapy: from molecular targets to clinical applications. This integrative schematic presents the diverse therapeutic mechanisms of microbial lipids across multiple levels of cancer biology. The figure organizes the mechanisms into interconnected categories: (A) Direct cytotoxic effects including membrane disruption, ROS generation, and DNA damage; (B) cell signaling modulation showing inhibition of PI3K/Akt, MAPK/ERK, and NF‐κB pathways; (C) anti‐metastatic actions depicting MMP inhibition, E‐cadherin upregulation, and cytoskeletal reorganization preventing migration and invasion; (D) angiogenesis inhibition through VEGF suppression and endothelial cell dysfunction; (E) immune modulation illustrating M2 macrophage repolarization, NKT cell activation, and cytokine profile adjustment; (F) drug resistance reversal showing P‐glycoprotein inhibition and enhanced chemotherapeutic uptake; and (G) epigenetic modifications via HDAC inhibition and histone acetylation. The diagram emphasizes the synergistic potential of these mechanisms, suggesting that microbial lipids suggest the potential to modulate multiple hallmarks of cancer in preclinical models, thereby offering advantages over conventional monotherapies.

Moreover, these lipids could affect the tumor micro‐environment by modulating inflammatory response and cytokine production—events known to participate in drug resistance development [144]. It is important to distinguish between native microbial lipids, which are directly produced by microorganisms through fermentation or biosynthesis, and synthetic analogs that are chemically derived based on microbial lipid structures. While native microbial lipids (e.g., PUFAs, sphingolipids from microbial sources) are obtained directly from microbial cultures, synthetic analogs (e.g., Edelfosine, Miltefosine, Erufosine) are chemically synthesized compounds designed to mimic or enhance the biological activities of their natural counterparts. In this review, we explicitly label synthetic analogs in relevant tables and discussions to avoid confusion. Both categories are discussed due to their shared mechanistic pathways and therapeutic potential, but readers should note that clinical translation and regulatory considerations may differ between native and synthetic compounds. Through these complex actions, microbial lipids represent a strategic route to curb drug resistance, to re‐optimize anticancer drugs and to promote better clinical outcomes in cancer therapy (Table 9; Figure 4).

**Table 9:** Lipids classified by their role in overcoming drug resistance and their functions.

Lipid type	Subtype	Microbial source	Function	References
Alkyl phosphocholines (APCs)	Miltefosine	Synthetic analog (derived from phospholipid structures; not directly microbial‐derived)	Inhibition of cellular signaling pathways, induction of apoptosis, and increased sensitivity of cancer cells to chemotherapeutic drugs	[145]
	Prefosine	Synthetic analog (derived from phospholipid structures; not directly microbial‐derived)	Inhibition of cellular signaling pathways, induction of apoptosis, and increased sensitivity of cancer cells to chemotherapeutic drugs	[146]
	Erufosine	Synthetic analog (derived from phospholipid structures; not directly microbial‐derived)	Inhibition of cellular signaling pathways, induction of apoptosis, and increased sensitivity of cancer cells to radiotherapy	[147]
Fatty acids	Caprylic acid (Octanoic Acid)	Caprylic acid (Octanoic Acid): *Aspergillus flavus*, *Pseudomonas* sp	Inhibition of biofilm formation, increased biofilm permeability, and increased bacterial susceptibility to antibiotics	[148]
	Linoleic acid	Linoleic acid: *Mortierella alpina*	Inhibition of biofilm formation and increased bacterial susceptibility to antibiotics	[149]
	Oleic acid	Oleic Acid: *Aspergillus* sp	Increased bacterial susceptibility to antibiotics	[150]
Lipopeptides	Surfactin	Surfactin: *Bacillus subtilis*	Inhibition of growth and proliferation of drug‐resistant cancer cells, increased cellular drug uptake, and decreased cellular drug efflux	[151]

*Note:* Compounds labeled as ‘Synthetic analogs’ are chemically derived based on microbial lipid structures but are not directly extracted from microorganisms. Native microbial lipids are directly produced via fermentation. The inclusion of synthetic analogs in this table reflects their mechanistic relevance to microbial lipid research, though their clinical development pathways may differ.

### 2.11. Regulation of Immune Cells/Modulation of Immune Response

Microbial lipids, including PUFAs, sphingolipids (such as ceramides, sphingosine 1‐phosphate [S1P], and ceramide 1‐phosphate [C1P]), and phospholipids (such as phosphatidylserine), are important for the regulation of immune responses in the context of cancer, influencing the activity and function of various immune cells. These lipids gain or lose immunoregulatory effects and affect cancer progression and cancer therapy through interactions with immune cells, including sphingolipids and phospholipids [152].

Sphingolipids (ceramides, S1P and C1P) are critical components in the regulation of the immune system. The dysfunctional immune cells can be induced to undergo apoptosis by Ceramide thereby maintaining immune homeostasis. However, contrary to S1P, S1P is a potent signaling molecule that enhances immune cell survival, proliferation, and migration, including T cells and macrophages [153]. The metabolic context determines whether this differential role of sphingolipids supports or contributes to immune evasion by tumors. Immune modulation is also contributed by phospholipids. For example, phosphatidylserine is an ’eat me’ signal to macrophages involved in the resolution of inflammation and preventing chronic immune activation that is often associated with tumor progression [154].

In addition, microbial lipids modulate the cytokine production profiles of immune cells that define the inflammatory tumor microenvironment. They can modulate pro and antiinflammatory cytokines production, and therefore influence the recruitment and activation of different immune cells within the tumor milieu [155].

Microbial lipids can modulate immune responses and provide a mechanism by which they can either suppress tumor growth by enhancing antitumor immunity or promote tumor growth through immune suppression. Such understanding provides potential therapeutic avenues to exploit microbial lipids for cancer immunotherapy to enhance the immune system’s capacity to fight cancer [156] (Table 10).

**Table 10:** Lipid types classified by their role in immune cell modulation and their functions.

Lipid type	Subtype	Function	Microbial source	Cancer types	References
Lipopeptides	Surfactin	Activates natural killer T (NKT) cells by binding to the CD1d receptor on antigen‐presenting cells (APCs), resulting in cytokine release	*Bacillus subtilis*	Breast, Lung	[157]
Sphingolipids	Sphingomyelin (SM)	Reduces the immunosuppressive phenotype of M2 macrophages	*Pichia pastoris*	Ovarian, Melanoma	[158]
	Ceramide (Cer)	Increases apoptosis in cancer cells; reduces the immunosuppressive phenotype of M2 macrophages	*Saccharomyces cerevisiae*	Colorectal, Prostate	[159]
Fatty acids	Palmitic acid (PA)	Reduces the immunosuppressive phenotype of M2 macrophages and increases IL‐12 expression	*Aspergillus niger*	Liver, Gastric	[160]
	Docosahexaenoic acid (DHA)	Decreases IL‐10 secretion in M2 macrophages	*Schizochytrium* sp.	Pancreatic, Breast	[161]
	Oleic acid	Induces differentiation of bone marrow‐derived macrophages into an M2‐like phenotype	*Aspergillus oryzae*	Skin, Prostate	[162]

### 2.12. Guided/Targeted Delivery

Microbial lipids, including PUFAs, phospholipids, and sphingolipids (such as ceramide), offer promise for guided or targeted delivery of anticancer agents, increasing the specificity and efficacy of chemotherapy while minimizing systemic toxicity. The amphiphilic nature and biocompatibility of these lipids make them good candidates for the formulation of nanoparticle based drug delivery systems. One of the most widely studied microbial lipid based delivery systems is liposomes, which are vesicles composed of phospholipid bilayers [163]. These structures can protect hydrophilic and hydrophobic drugs from degradation and release them at a controlled rate at the target site. Liposomes have a surface that can be modified with targeting ligands, like antibodies, peptides, or small molecules that know specific markers over expressed on cancer cells and help you target the cancer [164].

Ceramide enriched liposomes have also been made using another microbial lipid, sphingolipids. Both ceramide and these liposomes are stable; ceramide also helps with their anticancer activity by promoting apoptosis in malignant cells [165]. In addition, the incorporation of sphingolipids into liposomal membranes may affect the lipid raft domains to promote the fusion of the liposomal membranes with the target cell membranes and increase the drug delivery [166]. Moreover, the microbial lipid derived nanoparticles can be engineered to respond to specific stimulus such as pH or enzyme presence, which will trigger release of the encapsulated drug at the tumor site. This responsive delivery system delivers the drug in a manner that optimizes its therapeutic effect while minimizing drug exposure to healthy tissue toxic effects [167].

Overall, the development of microbial lipid‐based delivery systems represents a major advance in precision oncology with exciting potential to improve the efficacy and produce better patient outcomes by delivering drugs with appropriate and provided doses tailored to a patient’s needs.

### 2.13. Microbial Lipid‐Based Proteolysis Targeting Chimera (m‐PROTAC)

m‐PROTACs represent an innovative approach to cancer therapeutics, leveraging the properties of microbial lipids—including PUFAs, phospholipids, and sphingolipids—to enhance the delivery and efficacy of PROTAC molecules [168]. Bifunctional PROTACs recruit an E3 ubiquitin ligase to a specific target protein, leading to its ubiquitination and subsequent proteasome mediated degradation. The design of PROTACs incorporating microbial lipids is intended to improve solubility, permeability, and targeted delivery, overcoming some of the limitations encountered in conventional PROTACs [169].

Conjugation of microbial lipids, such as phospholipids and sphingolipids, to PROTAC molecules results in lipid‐PROTAC conjugates that show enhanced cell membrane interaction and uptake. Passive diffusion of PROTACs across the lipid bilayer can be facilitated by this lipid modification, which may increase intracellular delivery, especially in cells with altered lipid metabolism and membrane properties, such as cancer cells [170].

In addition, these lipids possess amphiphilic nature and can form micelles or liposomes as carriers for PROTAC molecules. These lipid based carriers can protect PROTACs from enzymatic degradation and limit off target effects by releasing PROTACs in a controlled manner [171]. Furthermore, m‐PROTACs can be tethered to lipid structures, and moieties, including antibodies or ligands, can be targeted to direct the m‐PROTACs to specific cancer cells containing specific markers [172].

**Figure 5:**
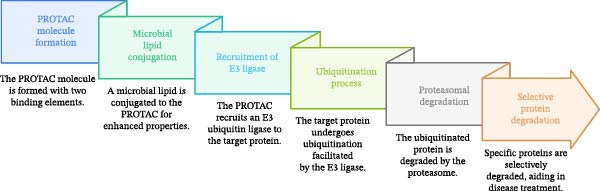
Mechanism of microbial lipid‐based proteolysis targeting chimera (m‐PROTAC) action in targeted cancer therapy. This figure illustrates the innovative m‐PROTAC technology that combines microbial lipid delivery systems with PROTAC molecules for enhanced cancer therapy. The diagram shows: (1) Formation of lipid‐PROTAC conjugates where microbial lipids (phospholipids, sphingolipids, or PUFAs) are covalently linked to bifunctional PROTAC molecules, (2) enhanced cellular uptake through passive diffusion across the lipid bilayer, facilitated by the amphiphilic nature of the lipid component, (3) targeted delivery to cancer cells via surface modification with ligands or antibodies recognizing tumor‐specific markers, (4) intracellular mechanism where the PROTAC molecule simultaneously binds to the target oncoprotein and an E3 ubiquitin ligase, forming a ternary complex, (5) ubiquitination of the target protein marking it for degradation, and (6) proteasome‐mediated destruction of the ubiquitinated oncoprotein. The figure highlights the advantages of m‐PROTACs over conventional PROTACs: improved solubility, enhanced membrane permeability, reduced off‐target effects through controlled release, and better therapeutic indices. This represents a promising precision oncology approach leveraging microbial lipid properties for next‐generation targeted protein degradation therapy.

Using microbial lipids, m‐PROTACs provide a promising way to target cancer with enhanced delivery, reduced systemic toxicity, and improved therapeutic indices. Although still largely conceptual and supported by early‐stage experimental research, m‐PROTAC technology highlights a potential intersection between lipid biology and targeted protein degradation strategies in oncology. Substantial preclinical validation is required before clinical applicability can be established [168] (Figure 5).

### 2.14. Safety, Toxicity, and Dosage Considerations

While microbial lipids, particularly PUFAs, exhibit significant therapeutic potential, their safety profile is dose‐dependent and context‐specific. It is crucial to acknowledge the paradoxical nature of PUFAs; while they act as antioxidants at physiological concentrations, high doses can exert pro‐oxidant effects, leading to lipid peroxidation and potential cytotoxicity in normal cells [173, 174]. Some studies have reported that excessive intake of PUFAs might promote carcinogenic effects under specific oxidative stress conditions. Therefore, establishing a therapeutic window is essential.

Current safety evaluation studies suggest that microbial lipids generally possess lower toxicity compared to conventional chemotherapy agents. However, adverse effects such as gastrointestinal discomfort, immune modulation risks, and interactions with other medications must be monitored. Future clinical trials should focus on defining the maximum tolerated dose (MTD) and identifying biomarkers for toxicity to ensure patient safety. Until robust clinical data is available, the use of microbial lipids should be approached with caution, emphasizing personalized dosage regimens.

In clinical nutrition studies, omega‐3 fatty acids such as EPA and DHA are commonly administered in ranges of 1–4 g/day in humans, depending on indication. However, these dosages are primarily evaluated in cardiovascular and inflammatory conditions rather than oncology [175, 176]. In cancer‐related studies, optimal dosing remains undefined and may differ substantially based on tumor type, formulation, and combination therapy. Therefore, extrapolation of nutritional doses to anticancer applications should be approached cautiously.

### 2.15. Limitations and Future Perspectives

Although microbial lipids demonstrate promising anticancer, antitumor, and antioxidant activities, several limitations must be acknowledged. First, the majority of available evidence derives from in vitro cell culture studies or in vivo animal models, with limited clinical trial data in human populations. The heterogeneity in experimental design, including variations in lipid concentrations, exposure duration, formulation strategies, and cancer models, makes direct comparison between studies challenging.

Second, the pharmacokinetic properties of many microbial lipids, including absorption, distribution, metabolism, and excretion (ADME), remain insufficiently characterized. Bioavailability, especially for highly unsaturated lipids, may vary depending on formulation and route of administration.

Third, the dual antioxidant and pro‐oxidant nature of PUFAs introduces complexity in determining optimal therapeutic windows. Dose‐dependent cytotoxicity toward normal cells and long‐term safety require systematic evaluation.

Future research should prioritize:

1. Standardized preclinical protocols,
2. Well‐designed randomized clinical trials,
3. Pharmacokinetic and pharmacodynamic profiling,
4. Comparative studies versus standard chemotherapeutics, and
5. Development of optimized delivery platforms.

Addressing these limitations will be critical for translating microbial lipid research from experimental models into clinically validated therapeutic strategies.

## 3. Production of Anticancer, Antitumor, and Antioxidant Microbial Lipids

Biomedical research and therapy increasingly involve the production of anticancer, antitumor, and antioxidant microbial lipids, including PUFAs, from various microorganisms such as bacteria, yeast, and fungi [177]. Fermentation optimization, genetic engineering, and metabolic pathway modification are applied to improve the yield and purity. With these strategies, the scalable and sustainable production of microbial lipids with defined therapeutic properties is enabled, enabling their clinical application for effective disease management and treatment [178].

### 3.1. Chemosynthesis Process

The chemosynthesis process, involving biosynthesis of anticancer, antitumor, and antioxidant microbial lipids, including PUFAs, utilizes chemical energy from the oxidation of inorganic substances [179]. As this method is particularly suitable for those environments where light for photosynthesis is not provided, this method has been used. Chemolithoautotrophic microorganisms use metabolic pathways specialized in the utilization of inorganic molecules such as hydrogen sulfide, ammonia, and methane as electron donors and inorganic carbon dioxide for an organic carbon fixation [180].

Chemosynthetic bacteria can be engineered to optimize the synthesis of specific lipid molecules with potent anticancer, antitumor and antioxidant properties in the context of lipid production. In this case, genetic engineering methods, for example, CRISPR‐Cas9 and TALEN, are used to change specific genes implicated in lipid pathway processing [181] (Table 11). Modifications of these lipids can lead to an increase in production of specific lipids or the addition of novel functionalities that improve their therapeutic efficacy [190]. This allows for scaling up of these processes in bioreactors under controlled conditions with parameters for temp, pH, and substrate concentrations exactly set in order to maximize lipid yield. The microbial lipids are extracted from the resultant using solvent extraction, centrifugation, or membrane filtration techniques [191].

**Table 11:** Comparison of CRISPR‐Cas9 and TALENs in lipid synthesis optimization.

Feature/technique	CRISPR‐Cas9	TALENs	References
Basic mechanism	Designs DNA‐binding proteins that can bind to specific DNA sequences, that will then be cut at those sequences	Designs DNA‐binding proteins that can bind to specific DNA sequences, that will then be cut at those sequences	[182]
Complexity of design	Relatively simple guide RNA design; does not require protein engineering	Requires custom protein engineering for each target sequence	[183]
Efficiency	Generally high efficiency and easy multiplexing capability	High efficiency but requires separate protein constructs	[184]
Off‐target effects	Off‐target effects Potential off‐target cleavage depending on guide RNA design	Generally lower off‐target risk due to longer recognition sequences	[185]
Cost	Lower cost due to simpler design and synthesis	Higher cost due to protein engineering requirements Scalability	[186
Applications in lipid synthesis optimization	Similar applications to CRISPR‐Cas9, though may be useful in cases where reducing off‐target cleavage is especially important	Similar applications to CRISPR‐Cas9, though may be useful in cases where reducing off‐target cleavage is especially important	[187]
Scalability	Highly scalable and adaptable for multiple targets	Less scalable due to custom protein desig	[188]
Flexibility	Limitations since new protein constructs need to be designed for each target deposited in database	Limitations since new protein constructs need to be designed for each target deposited in database	[189]

In addition to being a sustainable way to produce valuable therapeutic agents, chemosynthesis offers the opportunity to discover new lipid compounds that might not be easily obtained from natural sources by traditional extraction methods [192]. Such synthetic capability allows the fields of pharmacology and medicinal chemistry to push forward the development of compound classes under the framework of new drugs and therapies.

#### 3.1.1. Solution‐Phase Lipid Synthesis

Solution‐phase lipid synthesis is crucial for producing bioactive microbial lipids, including PUFAs, with anticancer, antitumor, and antioxidant properties [193]. In this type, lipids are synthesized and the reagents and products of the lipids are dissolved in solutions to undergo the reaction processes of lipid assembly. The approach is especially useful for synthesizing multilayer lipid structures that are challenging to biosynthesize or isolate from natural sources [194]. As in solution‐phase synthesis, the lipid molecules are built gradually with one functional group at a time. This makes it possible to manipulate the molecular size and shape of the lipids, besides being able to introduce functional groups that improve the biological performance of the lipids [195]. Speaking of solvents and catalysts, the choice depends on their efficiency in conditions of improved reaction yield. Chloroform, methanol, and dichloromethane solvents are preferred mainly because they dissolve lipid components nicely and most of the catalysts employed in esterification and acylation reactions [196].

The process uses a variety of technologies like carbodiimide mediated activation for the purpose of nucleophilic concentration for stimuli reactive assembly for ester links, which are important in lipid synthesis [197]. Further, there is often necessity to protect reactive groups not to undergo unwanted transformations, they are then removed by deprotection [198]. Solution‐phase synthesis is also quantitative, which would conform nicely to synthesizing a copious number of lipid compounds [199]. But to use it effectively and safely, especially in dealing with organic solvents and by‐products, then the company must follow environmental and safety laws [200]. This method can be useful in the design of new lipid base therapeutics since it allows synthesis of different lipid structures that may be beneficial in cancer treatment and/or prevention [201].

#### 3.1.2. Solid‐Phase Lipid Synthesis

Solid‐phase lipid synthesis, an increasingly important method for producing complex lipid structures with biological activities such as anticancer, antitumor, and antioxidant properties, including PUFAs, involves the stepwise coupling of lipid precursors onto a beaded matrix [202]. This method entails the stepwise coupling of lipid precursors onto a beaded matrix and the advantage it posses is that the synthesis is hierarchical and easily purifiable. In solid‐phase synthesis the first lipid fragment is attached to a resin and this is the solid phase support. Further lipid precursors are then assembled sequentially by similar reactions which include well established coupling reagents and protection, deprotection methods to improve the selectivity and outcome of the reactions [203]. Every addition is accompanied by washing steps to drive out unreacted reactants and side products, a feature that is advantageous over solution‐phase synthesis since no additional purification is required once the synthesis is through [204].

Another reason why solid support is employed enables the semi‐automated or fully automated synthesis process which enables a huge increase in the scale and yield of lipid. This is especially the case in generating lipid libraries essential in drug screen and functional analysis [205]. Furthermore, solid‐phase synthesis enables attachment of functional groups that can boost the affinity that the lipid exhibits with a certain target within a cell, making the lipid either a more effective agent at killing cancer cells by destroying their outer membrane or at controlling immune responses [206]. One of the major limitations of solid‐phase lipid synthesis is the issue of possible replication length, which can be a limitation to the length of the lipid chain that can be synthesized. Despite certain limitations, new linker chemistry and improved solid support push this method forward and place it among the most efficient strategies for the synthesis of bioactive lipids for medicine [207].

### 3.2. Biosynthesis Processes

Microbial lipid synthesis techniques, including biosynthesis methods for producing anticancer, antitumor, and antioxidant microbial lipids such as PUFAs, leverage the metabolic capabilities of microorganisms to create highly complex lipids [208]. These processes are in fact necessary for formation of bioactive products that are potentially useful in pharmacy. Microbial biosynthesis is preferred for its environmental friendly approach in that it uses renewable resources and commonly consumes less energy than chemical synthesis [209]. These naturally occurring microorganisms include bacteria, yeast, and fungi where metabolic pathways of the engineered microorganisms dictate the synthesis of the required lipids [210]. This is done using genetic engineering technology where boosts genes responsible for the synthesis of the desired lipids are over expressed or else the metabolic routes that compete with the specific lipids are locked in place to allow the flow of intermediates to be directed towards the lipids of interest.

The most frequently applied system is the fatty acid synthase (FAS) systems of microorganisms providing for chain elongation in lipid formation. It has been elucidated that when the expression of enzymes within the FAS pathway is modulated, it is possible to increase the synthesis of fatty acids which in turn forms the building blocks of other lipids [211].

Furthermore, the microorganisms’ fermentation requirements such as nutrient medium, pH, temperature, and aeration are also enhanced to enhance lipid productivity and characteristics [212]. This biosynthetic approach does not only provide the synthesis of a certain lipid molecules with anticancer and antioxidant properties but also produce new lipid compounds have never been found in nature [213].

With the help of new genetic and bioprocessing results, biosynthesis processes are occupied by improving the yield and functionality of microbial lipids to be applicable for therapeutic uses in combating cancer and related diseases.

#### 3.2.1. Enzymatic Modification

Enzymatic modification significantly enhances the anticancer, antitumor, and antioxidant activity of microbial lipids, including PUFAs, by altering their structure and properties to improve their therapeutic potential [25]. The enzymatic approach is preferred because it is selective, rapid, and requires low temperatures such that the structural integrity of complex lipids is not compromised.

Lipases and phospholipases are widely used enzymes in the alteration of microbial lipids. These enzymes participate in ester bond cleavage in lipids, the process by which fatty acids can be added or released from a lipid. This change can greatly affect the physical and chemical characteristics including melting point and solubility that are characterized the lipid and its ability to interact with cancer cells membranes for biological activity [214].

Furthermore, the functional groups may be introduced into lipid structures with the help of enzymes also acting as catalysts. These changes may increase the ability of the molecule to produce ROS or to modulate immune factors; the latter of these is an action by which lipids may achieve anticancer activity. For instance, the rebuilding of the lipid structures with PUFAs by enzymatic catalysis was reported to alter cancer cell membranes, thus causing apoptosis [215].

Enzymatic modification is not limited to functionalization but also encompasses regioselective as well as stereoselective modification of lipids for specific lipid molecules with the prospect of such therapeutically related applications [216]. Apart from enhancing the performance of lipid based therapies, this precision also reduces the likelihood of side effects paving enzymatic modification as vital in the advancement of lipid based anticancer agents (Table 12).

**Table 12:** Enzymes used in enzymatic modification of lipids and their applications.

Enzyme type	Specific enzyme	Microbial source	Applications	References
Lipases	Pancreatic lipase	*Porcine pancreas*	‐ Hydrolyzing fats into fatty acids and glycerol	[217]
			‐ Used in the dairy industry for flavor development in cheeses	
	Candida lipase	*Candida antarctica*	‐ Biodiesel production through transesterification of oils	[218]
			‐ Processing fats in food products	
	Rhizopus lipase	*Rhizopus oryzae*	‐ Synthesis of structured lipids	[219]
			‐ Used in oleochemical industries for modification of fats and oils	
Phospholipases	Phospholipase A1	*Aspergillus niger*	‐ Used to release fatty acids from phospholipids	[220]
			‐ Production of lysophospholipids for food emulsifiers	
	Phospholipase A2	*Streptomyces violaceoruber*	‐ Extraction of yolk lecithin	[221]
			‐ Used in medical applications to produce anti‐inflammatory agents	
	Phospholipase C	*Bacillus cereus*	‐ Used in the pharmaceutical industry to produce second messengers in signal transduction	[222]
	Phospholipase D	*Streptomyces chromofuscus*	‐ Production of phosphatidic acid in biotechnological applications	[223]
			‐ Used in food industry to improve dough	

#### 3.2.2. Genetic Engineering Techniques

Genetic engineering techniques, including CRISPR‐Cas9, RNA interference (RNAi), and plasmid‐based gene transfer, are crucial for enhancing microbial lipid production, particularly of PUFAs with anticancer, antitumor, and antioxidant properties [224]. The most used techniques in this class of work include CRISPR‐Cas9, RNAi and utilization of plasmid for gene overexpression or suppression. CRISPR‐Cas9 is the state‐of‐the‐art technique for genome editing which enables deletions, insertions and modifications of genes on microbial DNA, in case of lipid metabolic genes [225]. Such accuracy allows for the redirection of metabolic flux towards the generation of desired lipid compounds for treatment of cancer [226]. For example, by targeting genes that encode for proteins involved in omega‐3 fatty acid biosynthesis, the amounts of these healthy anti‐inflammatory and anticancer lipids in microbial cells can be enhanced.

Another method of gene expression discovered in microorganisms is called RNAi. Using siRNA one is able to selectively knock down certain undesirable genes resulting in lowered synthesis of undesired lipids and, at the same time, enhanced production of the lipids with anticancer potential [227].

Also, there is is the use of plasmid vectors to transfer new biosynthetic capabilities to existing microorganisms during genetic engineering. With transfer and introduction of such genes originating from other organisms that harbor the genes promoting synthesis of enzymes leading to the production of diverse lipids, researchers and scientists can enhance the production of a more diverse bioactive lipids by the microbial hosts [228].

By using these genetic engineering approaches, the microbial strains can be designed to produce lipid molecules that, in term, could be delivered to cancers selectively and with maximal efficiency, thus increasing the potential of microbial‐derived lipid therapies for cancers [224] (Table 13).

**Table 13:** Comparison of genetic engineering techniques in microbial lipid production.

Technique	Mechanism	Applications in microbial lipid production	References
CRISPR‐Cas9	Utilizes a guide RNA to direct Cas9 to specific DNA sequences for gene editing	‐ Precision editing of genes involved in lipid metabolism	[229]
		‐ Knockout of genes that repress lipid accumulation	
		‐ Enhancement of pathways to increase lipid content and profile	
RNAi	Uses small interfering RNA (siRNA) molecules to inhibit gene expression through the degradation of mRNA	‐ Downregulation of genes that negatively affect lipid synthesis	[230]
		‐ Used to study gene function by selectively silencing genes	
Plasmid use	Involves the introduction of plasmids carrying specific genes into microbial cells to alter their genetic makeup	‐ Overexpression of beneficial genes to increase lipid production	[231
		‐ Introduction of new metabolic pathways to diversify lipid types	

## 4. Clinical Application of Anticancer, Antitumor, and Antioxidant Microbial Lipids

Microbial lipids, including PUFAs like omega‐3 fatty acids, and other bioactive lipids such as sphingolipids and phospholipids, are showing significant promise in oncology due to their anticancer, antitumor, and antioxidant properties [232, 233]. Sphingolipids, fatty acids and phospholipids are some of the microbial lipids reported to possess strong biological activities such as apotopsis, antiproliferative, and free radical scavengers [234]. Certain microbial‐derived lipids, particularly omega‐3 fatty acids, are currently used as adjunctive nutritional supplements in clinical settings and are being investigated for supportive roles alongside conventional therapies. For instance, the omega‐3 fatty acids originating from marine microalgae have been seen to overcome negative interferences of the disease and improve the chance of the treatment’s success by making tumors more susceptible to chemotherapy. Several clinical studies have also shown that these fatty acids can slow the growth of tumors and enhance patient’s survival rates [232] (Table 14).

**Table 14:** Lipids with clinical application as anticancer, antitumor, and antioxidant agents and their mechanisms of action.

Lipid type	Sub‐category	Mechanism of action	Microbial source	References
Fatty acids	Omega‐3	Induction of apoptosis in cancer cells; Inhibition of tumor signaling pathways; Enhancement of chemotherapy drug efficacy	*Schizochytrium* sp., *Crypthecodinium cohnii*	[235]
	Omega‐6	Precursor to eicosanoids (potent regulators of tumor cell proliferation and death)	*Mortierella alpine*, *Arthrospira platensis*	[236]
	Saturated fatty acids	Modulation of tumor cell survival	*Corynebacterium glutamicum*	[237]
Alkylphospholipids (ALPs)	—	Modulation of tumor cell membrane function; Interference with phospholipid homeostasis; Induction of apoptosis in tumor cells; Significant activity against skin cancer and some leukemias	*Candida albicans*	[238]
Ceramides	—	Modulation of tumor cell survival	*Saccharomyces cerevisiae*, *Aspergillus niger*	[239]
Other lipids	PA	Reduction of the immunosuppressive phenotype of M2 macrophages; Induction of apoptosis in cancer cells	*Bacillus subtilis*, *Rhizopus oryzae*	[240]
	SM	Reduction of the immunosuppressive phenotype of M2 macrophages	*Pichia pastoris*, *Aspergillus niger*	[241]
	Cer	Reduction of the immunosuppressive phenotype of M2 macrophages	*Saccharomyces cerevisiae*	[242]
	DHA	Reduction of the immunosuppressive phenotype of M2 macrophages	*Schizochytrium* sp	[243]
Lysophosphatidylcholine analogs	—	Inhibition of tumor cell proliferation; Induction of apoptosis in tumor cells; Inhibition of tumor metastasis; Inhibition of angiogenesis; Differentiation of tumor cells	*Pseudomonas aeruginosa*	[244]

Furthermore, some of the microbial sphingolipids have been taken to clinical trials that are in the acting as single agents against cancer. These compounds can trigger apoptosis of malignant cells without affecting the healthy cells in the body, and that kind of selective toxicity gives this method a tremendous advantage over conventional chemotherapy. The mechanism requires the alteration of some critical pathways including, cell cycle and apoptotic signaling [245].

The putative antioxidant microbial lipids are also being studied for their efficacy as shields against the oxygen toxicity, a prevalent side effect of many cancer therapies [246]. These lipids do not only secure normal cells from oxidation in chemotherapy but also prevent carcinogenesis and cancer development.

Studies into the efficacy of microbial lipids advance, while the application of these lipids in clinical practice extends—with more trials continuing on the identification of the most effective dosing regimens, modes of administration, and microbial lipid associations with other treatments [247]. This integration demonstrates the power of microbial lipids as extra‐ and main‐stream supportive and standalone treatments in cancer management plans, and advances the state of the art in individualized medicine and patients’ welfare.

## 5. Conclusion

The importance of PUFAs, a new generation of anticancer, antitumor, and antioxidant compounds, is highlighted in this review. The action of PUFAs in cancer treatment has been shown to be multifaceted, including membrane disruption, induction of apoptosis/necrosis, DNA damage, histone modification, angiogenesis inhibition, cell proliferation/cycle regulation, migration, invasion and metastasis inhibition, differentiation induction, reversal of drug resistance, and modulation of immune system. Advantageously, they are also economical biosynthesized through microbial fermentation, chemosynthesis, and other synthesis techniques.

Further clinical confirmation is necessary, but PUFAs represent a promising strategy for cancer management throughout the world, especially in developing countries where conventional therapies are more toxic and less accessible. They review their diverse mechanisms of action, and their potential for more targeted drug delivery using these lipids. This, however, points out that more research and clinical trials are required to fully test their efficiency and safety in human populations to establish pharmaceutically acceptable formulations for administration (capsules, injections or topical solutions). They are a potential new strategy for global cancer therapy, particularly in low resource settings, based on the diversity of their mechanisms of action, potential for low cost production, and potentially low toxicity. These microbial derived substances have potential to be therapeutically exploited further, and this further research is necessary.

In summary, microbial lipids represent an emerging and biologically versatile class of bioactive molecules with potential applications in oncology. While current evidence supports their multifaceted mechanistic roles in experimental systems, further translational and clinical studies are required before definitive therapeutic conclusions can be drawn.

## Author Contributions

**Morteza Nadeb and Tahereh Komeili-Movahed**: wrote the manuscript, edited the manuscript. **Seyed Soheil Aghaei and Hamed Afkhami**: design and supervision.

## Funding

No funding was received for this manuscript.

## Disclosure

All authors read and approved the final manuscript.

## Conflicts of Interest

The authors declare no conflicts of interest.

## Data Availability

The data that support the findings of this study are available from the corresponding author upon reasonable request.
